# Plasma HSP90AA1 Predicts the Risk of Breast Cancer Onset and Distant Metastasis

**DOI:** 10.3389/fcell.2021.639596

**Published:** 2021-05-24

**Authors:** Haizhou Liu, Zihan Zhang, Yi Huang, Wene Wei, Shufang Ning, Jilin Li, Xinqiang Liang, Kaisheng Liu, Litu Zhang

**Affiliations:** ^1^Department of Research, Affiliated Tumor Hospital, Guangxi Medical University, Nanning, China; ^2^Shenzhen People’s Hospital (The Second Clinical Medical College, Jinan University, The First Affiliated Hospital, Southern University of Science and Technology), Shenzhen, China

**Keywords:** breast cancer, metastasis, heat shock protein-90, prognosis, nomogram

## Abstract

**Aim:**

We aimed to develop and validate a comprehensive nomogram containing pre-treatment plasma HSP90AA1 to predict the risk of breast cancer onset and metastasis.

**Methods:**

We assessed the expression of HSP90s in breast cancer patients using an online database. To verify the results, 677 patients diagnosed with breast cancer and 146 patients with benign breast disease between 2014 and 2019 were selected from our hospital and were divided into cancer risk and metastasis risk cohorts. We focused on HSP90AA1 to elucidate the risks of onset and metastasis in the cohorts.

**Results:**

Expression levels of *HSP90AA1*, *HSP90AA2*, *HSP90AB1*, *HSP90B1*, and *TRAP1* were linked to disease progression. Survival analysis using the GEPIA and OncoLnc databases indicated that the upregulation of *HSP90AA1* and *HSP90AB1* was related to poor overall survival. In the cancer risk cohort, carcinoembryonic antigen (CEA), carbohydrate antigen 153 (CA153), HSP90AA1, T cells%, natural killer cells%, B cells%, neutrophil count, monocyte count, and d-dimer were incorporated into the nomogram. A high Harrell’s concordance index (C-index) value of 0.771 [95% confidence interval (CI), 0.725–0.817] could still be reached in the interval validation. In the metastasis risk cohort, predictors contained in the prediction nomogram included the use of CEA, CA153, HSP90AA1, carbohydrate antigen 125 (CA125), natural killer cells%, B cells%, platelet count, monocyte count, and d-dimer. The C-index was 0.844 (95% CI, 0.801–0.887) and it was well-calibrated. HSP90AA1 raised net clinical benefit of breast cancer onset and metastasis risk prediction nomogram in a range of risk thresholds (5–92%) and (1–90%).

**Conclusion:**

Our study revealed that pretreatment plasma HSP90AA1 combined with other markers could conveniently predict the risk of breast cancer onset and metastasis.

## Introduction

Breast cancer is one of the most common malignant tumors worldwide, with high incidence and mortality rates. It is the most frequent cancer and the second leading cause of cancer-related death in females (Bray et al., 2018; Siegel et al., 2018). Surgery, endocrine therapy, and chemotherapy are the primary treatment approaches for breast cancer, and breast cancer that is detected early-on always has a better prognosis after surgery. The 5-year survival rate of early stage breast cancer patients is estimated to be as high as 90%, and declines to 27% once metastasis has occurred (Siegel et al., 2018). Early and timely diagnosis is crucial for the treatment and prognosis of breast cancer patients. Hence, it is urgent to explore non-invasive diagnostic biomarkers that can allow clinicians to distinguish between benign and malignant tumors as well as to detect the stage of cancer in order to develop an individualized treatment plan.

Heat shock protein 90-alpha (HSP90AA1), an essential molecular chaperon that is highly conserved in evolution, is efficiently expressed under the stimulatory conditions of trauma, infection, and tumors. Newly formed HSP90AA1 can be secreted into the extracellular environment and can also enter the nucleus to stimulate the formation of immune memory and participate in tumor formation (Frydman, 2001; Condelli et al., 2019). HSP90AA1 plays an essential role in DNA damage regulation, cell cycle regulation, gene expression, and carcinogenesis (Condelli et al., 2019). Previous research has demonstrated that in cancer cells, HSP90AA1 activates many oncogenic client proteins, thereby stimulating cell survival, growth, and invasiveness (Eustace et al., 2004; Chehab et al., 2015; Wu et al., 2017). These results suggest that HSP90AA1 could serve as a biomarker for cancer. Furthermore, many experiments have shown that HSP90AA1 is able to promote cancer cell proliferation, metastasis, invasion, and epithelial-to-mesenchymal transition in several diseases, suggesting that HSP90AA1 could be a potential target for the treatment of cancers (Wu et al., 2017; Tian et al., 2019).

Biomarkers in peripheral blood are considered widely accepted and convenient predictors of cancer. Carbohydrate antigen 153 (CA153) is now regarded as the most critical tumor marker for breast cancer, although its sensitivity and specificity are not sufficiently high. Recently, some studies have indicated that overexpression of HSP90AA1 is related to various malignant tumors. For example, previous studies have confirmed that HSP90AA1 is a sensitive biomarker of lung cancer and is valuable in predicting the response of lung cancer patients to surgery or chemotherapy (Shi et al., 2014). It can also be used as an initial diagnosis in patients with hepatocellular carcinoma (Fu et al., 2017; Wei et al., 2020). Other studies have reported that high expression of HSP90AA1 is associated with poor prognosis in patients with colorectal cancer (Zhang et al., 2019). In a multi-center clinical study of 1,558 patients (39 cases of breast cancer), plasma HSP90AA1 can be used as a biomarker for cancer (Liu et al., 2019). It has been reported that elevated plasma HSP90AA1 levels are specific to malignant tumors and patients with metastatic liver or breast tumors have higher plasma HSP90AA1 levels than those without metastasis (Wang et al., 2009). Thus, HSP90AA1 is considered a potential tumor biomarker, but the utility of measuring plasma HSP90AA1 in breast cancer clinical treatment has not yet been investigated. Nomogram is a common tool in oncology and medicine; one of its main advantages is the ability to estimate individual risk based on patient and disease characteristics (Balachandran et al., 2015). In this study, we aimed to examine the diagnostic value of plasma HSP90AA1 in breast cancer by using an online database and clinical parameters. We also developed prediction nomogram models for assessing the risk of breast cancer and breast cancer metastasis.

## Materials and Methods

### Bioinformatics Data Mining

The online tool “ONCOMINE”^1^ was used to evaluate the expression patterns of HSP90 family genes (HSP90s) in different cancers. Further, the online tool “Gene Expression Profiling Interactive Analysis” (GEPIA)^2^ was used to draw the correlation between the expression of HSP90s and overall survival (OS). “UALCAN”^3^ was used toextract the tumor grade in the Cancer Genome Atlas (TCGA) breast cancer dataset. We used mass spectrometry data in the Human Protein Atlas (THPA)^4^ to analyze the expression of HSP90 gene-encoded proteins in patient plasma samples.

### Selection of Breast Cancer Patients

The participants in this retrospective study were selected from the Guangxi Medical University Cancer Hospital between May 2014 and August 2019. The inclusion criteria were as follows: (1) women with primary breast cancer or breast benign disease; (2) breast cancer was confirmed histologically; and (3) patients whose clinical characteristics were complete. The exclusion criteria were as follows: (1) patients with more than one malignant tumor in the same period; (2) patients with severe systemic infection, acute or chronic hematologic or autoimmune diseases; and (3) incomplete information on any of these clinical characteristics. This study followed the ethical guidelines of the 2008 Declaration of Helsinki and was approved by the Ethics Committee of Guangxi Medical University Cancer Hospital (LW2020065).

### Clinical and Hematology Test Data Collection

In this retrospective study, the data on the clinical-pathological features and biomarkers in the peripheral blood of the selected patients diagnosed with breast disease were collected. These included carcinoembryonic antigen (CEA), carbohydrate antigen 125 (CA125), carbohydrate antigen 153 (CA153), HSP90AA1, d-dimer, estrogen receptor (ER), progesterone receptor (PR), human epidermal growth factor receptor-2 (HER2), Ki-67, CK5-6, epidermal growth factor receptor (EGFR), TNM stage, tumor location, and tumor metastasis sites. Based on the previous studies, we assumed that tumor occurrence and prognosis may be related to cellular immunity (T cells, helper T cells (Th), killer T cells (Tc), natural killer (NK) cells, B cells, neutrophils, monocytes, and platelets (Liu et al., 2016; Tang et al., 2020). Therefore, this article also included cellular immunity-related indicators. All blood samples were collected before treatment.

### Clinical Molecular Typing

The division of hormone receptor (HR) negative/positive patients: either estrogen receptor (ER) or progesterone receptor (PR) positive or double-positive is HR-positive (528 cases), and double-negative is HR negative (149 cases). Human epidermal growth factor receptor-2 (HER2) negative/positive expression classifies patients as HER2-positive/negative (347/330 cases).

### Assessment of HSP90AA1 Levels

The levels of plasma HSP90AA1 were measured using an ELISA kit for HSP90AA1 protein (Yantai Protgen Biotechnology Development Co., Ltd., Yantai, China). Fresh blood samples (2 mL) were collected from patients and controls and were combined with EDTA-K2 anticoagulant. All steps were performed according to the manufacturer’s instructions. The fresh blood samples were first preincubated at 37°C for 30 min, then centrifuged at 3,000 rpm for 10 min, and diluted 20 times with the diluent solution provided. Then, the standards were loaded together with the quality controls, and the prepared samples (50 μL of each) were added to 96-well plates followed by the addition of 50 μL of the anti–HSP90AA1HRP-conjugated antibody. These were incubated at 37°C, and samples were subjected to gentle shaking for 1 h. Next, the plates were washed six times using the washing buffer provided in this kit, which was followed by the chromogenic reaction; 50 μL of the peroxide and 50 μL of 3, 3, 5, 5′-tetramethylbenzidine were added the samples, which were then incubated at 37°C for 20 min. The reaction was terminated by the addition of an acid stop buffer. Finally, the optical density was measured using a spectrophotometer using an excitation of 450 nm and a detection wavelength of 620 nm as the reference wavelength. The concentration of HSP90AA1 protein in each sample was calculated according to a standard curve of the optical density values.

The levels of plasma HSP90AA1 were measured using Western blotting. Erasin of abundance proteins, including immunoglobulin G (IgG) and albumin, from plasma samples, was performed using Spin Albumin and IgG Erasin Kit [Sangon Biotech (Shanghai) Co., Ltd.] according to the manufacturer’s instructions. Next, Put a spin column into a 2 mL collection tube. Turn the reagent bottle containing the resin upside down, mix well to form a uniform suspension, and add 340 μL of the suspension to the spin column. Centrifuge at 7,500 rpm (10,000 g) for 1 min, pour out the liquid in the collection tube and then put the resin-containing spin column into the collection tube again. Add 300 μL of binding/washing solution to the spin column, close the lid, turn upside down 3∼5 times or vortex for 10 s, then centrifuge at 7,500 rpm (10,000 g) for 1 min and pour out the liquid in the collection tube. Repeat this step 2∼3 times until the liquid flowing out is almost colorless. Take a clean centrifuge tube and add 10 μL of plasma sample to 200 μL of pre-chilled binding/washing solution and mix well. Add the above mixture to the spin column containing the resin, close the lid, invert 3– 5 times, or vortex for 10 s. Shake on a shaker at 4°C at medium speed for 15 min or vortex for 15 min. Then transfer to a refrigerated centrifuge, centrifuge at 7,500 rpm (10,000 g) at 4°C for 1 min, and collect the filtrate. Transfer the filtrate in the collection tube to the spin column containing the resin again, and vortex for 15 min at 4°C on a shaker or vortex for 10–15 min. Transfer to a refrigerated centrifuge, centrifuge at 7,500 rpm (10,000 g) for 1 min at 4°C, and add 200 μL of pre-cooled binding/washing solution to the spin column containing the resin. Centrifuge at 4°C at 7,500 rpm (10,000 g) for 1 min. At this time, the collection tube contains about 400 μL of filtrate. This filtrate is a sample from which plasma albumin and IgG antibodies have been removed. Samples mixed with loading buffer were denatured at 95°C for 5 min and separated on SDS−PAGE gels as previously described. Separated on 8% SDS−PAGE gels, followed by transfer to Nitrocellulose membrane (NC). The target proteins were probed with the indicated primary antibodies (Cat no: 13171-1-AP, Proteintech Group, Inc.) overnight at 4°C, then incubated with horseradish peroxidase (HRP)−conjugated secondary antibodies (Abcam (Shanghai) Trading Co., Ltd.) for 1 h at room temperature, and visualized using a common enhanced chemiluminescence technique (Abcam) according to the manufacturer’s protocol.

### Construction of the Nomograms

The least absolute shrinkage and selection operator (LASSO) regression model was used to select the relevant factors of breast cancer diagnosis and metastasis. The logistic regression analysis was then used to establish a convincing prediction model for cancer risk and metastasis risk by merging the features selected in the LASSO method. Clinicopathological features and biomarkers in the peripheral blood with a *p*-value of less than 0.05 were included in the model. All selected predictors were used to develop a predictive nomogram model for cancer risk and metastasis risk in breast cancer patients.

### Validation and Calibration

The nomograms were subjected to 1,000 bootstrap resamples for internal validation of the two cohorts. The concordance index (C-index) between the predicted probability and response was used to assess the discrimination performance of the nomograms (Wolbers et al., 2009). The value of the C-index ranges from 0.5 to 1.0, with 0.5 indicating random chance and 1.0 indicating perfectly corrected discrimination (Pencina et al., 2008). Calibration is the ability of a model to make unbiased estimates of the outcome. The marginal estimate versus the average predictive probability of the models was used to construct calibration curves. For a well-calibrated model, the predictions were expected to fall on a 45° diagonal line.

### Statistical Analyses

Statistical analyses were performed using R statistical software (version 4.0.2). Differences between discrete variables were examined by box plots. Receiver operating characteristic (ROC) curves were established to determine the optimal cut-off thresholds and diagnostic accuracies of the continuous variables. *P*-values (all two-sided) less than 0.05 were considered statistically significant.

## Results

### HSP90AA1 Is More Suitable as a Potential Clinical Target and Prognostic Biomarker

**FIGURE 1:**
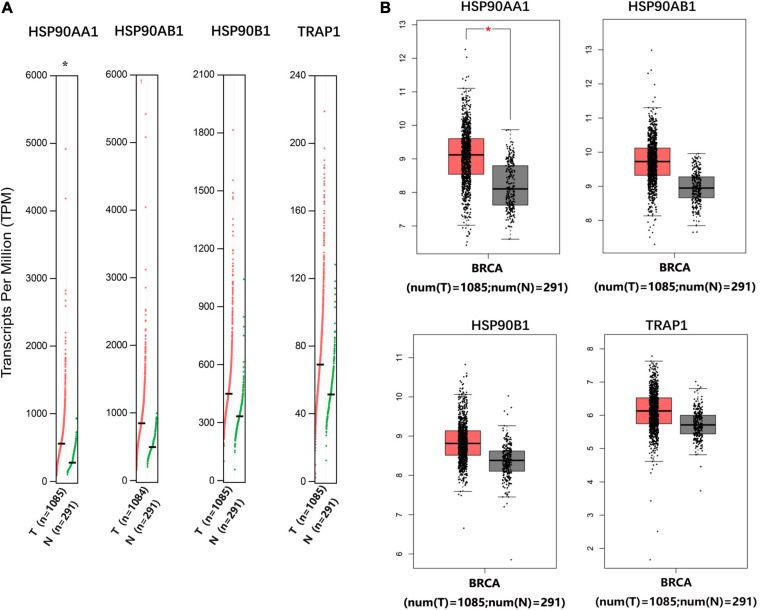
Gene Expression Profiling Interactive Analysis of the expression profiles of HSP90AA1, HSP90AB1, HSP90B1, and TRAP1 in breast cancer patients. The profile shows the tissue-wise expression of heat shock protein 90 kDa family gene signatures in breast cancer using **(A)** a dot plot and **(B)** a box plot match of TCGA normal and GTEx data. *Indicates a statistically significant difference (*p* < 0.05).

**FIGURE 2:**
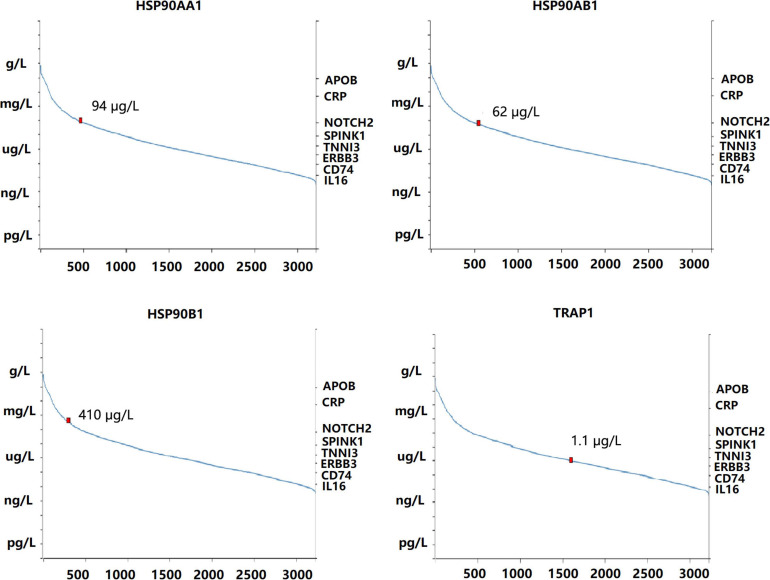
Concentration of heat shock protein 90 in the blood quantified by mass spectrometry-based plasma proteomics.

The mRNA levels of *HSP90AA1* in breast cancer tissues were higher than those in normal tissues (Figures 1A,B). Although the mRNA levels of *HSP90AB1*, *HSP90B1*, and *TRAP1* were increased in breast cancer tissues compared with those in normal tissues, the mRNA levels were not significant. We further used Oncomine to verify these findings (Supplementary Figure 1). The UALCAN results suggested that advanced breast cancer patients were more inclined to show higher HSP90s expression levels (Supplementary Figure 2A). Further, the GEPIA results revealed that increased expression levels of *HSP90AA1* and *HSP90AB1* were strongly associated with poor OS (Supplementary Figure 2B). HSP90s protein target was detected in plasma for the reference set (Peptide atlas) using mass spectrometry; the protein expression level of HSP90AA1 in the plasma was 94 μg/L (Figure 2).

### Clinical Characteristics

**FIGURE 3:**
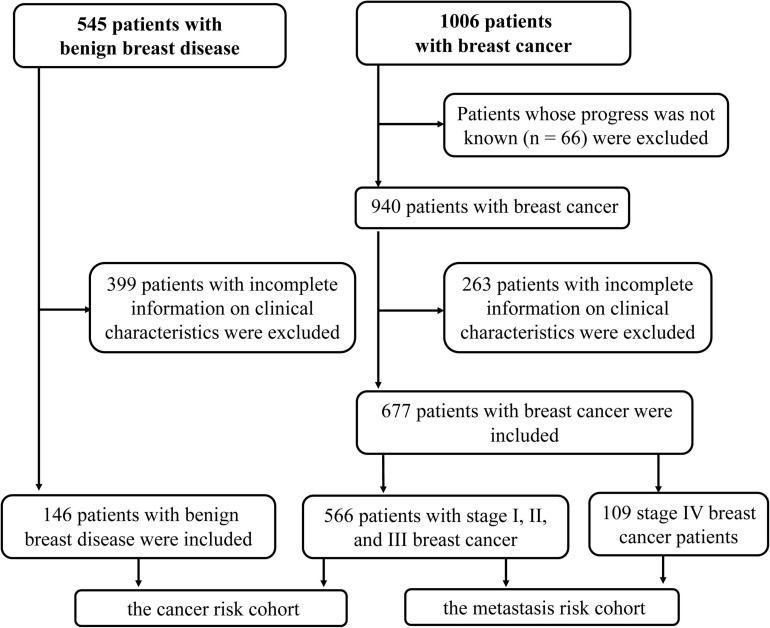
Schematic diagram of patient enrollment in this study.

**FIGURE 4:**
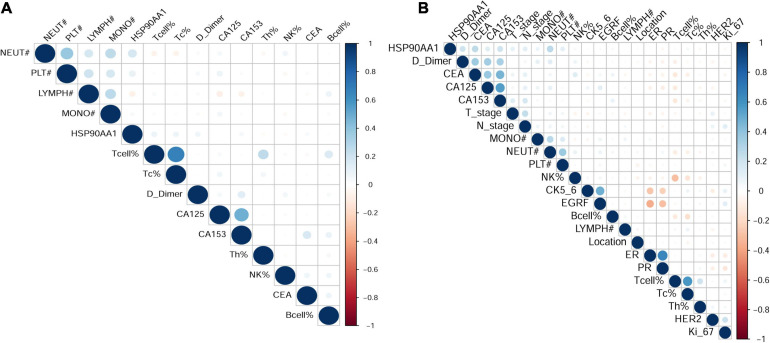
Visualization of the correlation matrix of all clinical biomarkers including HSP90AA1. **(A)** Benign breast disease and stage I-III breast cancer; **(B)** breast cancer stages I-III and IV.

In order to verify the above results and analyze the expression pattern of HSP90AA1 in breast cancer, a total of 677 breast cancer patients and 146 breast benign disease patients were selected after applying the inclusion and exclusion criteria, and all enrolled patients were female. We selected 146 patients with benign breast disease and 566 patients with stage I, II, and III breast cancer as the cancer risk cohort. Among breast cancer patients, we included 566 stage I-III patients and 109 stage IV patients as the metastasis risk cohort (Figure 3). The characteristics of these patients are summarized in Table 1. We visualized the correlation matrix of all clinical biomarkers in the two cohorts. There was no significant correlation between the expression of plasma HSP90AA1 and any of the clinical indicators (Figure 4).

**TABLE 1:** Patient characteristics.

Characteristic	Breast cancer	Benign breast disease
Number of patients	677	146
**Age (years)**		
Average (SD)	50 (10.97)	42 (12.34)
Median (IQR)	49 (42.56)	43 (32.50)
<45 years (%)	223 (32.9%)	85 (58.2%)
≥45 to <55 years (%)	247 (36.5%)	42 (28.8%)
≥55 years (%)	207 (30.6%)	19 (13.0%)
**T**		
T_1_T_2_	511 (75.5%)	/
T_3_T_4_	166 (24.5%)	/
**N**		
N_0_	322 (47.6%)	/
N_1__–__*X*_	355 (52.4%)	/
**M**		
M_0_	567 (63.2%)	/
M_1_	110 (36.8%)	/
**Installment**		
I/II	428	/
III/IV	249	/
**Predictive factors**		
Progesterone receptor + (%)	465 (68.7%)	/
Estrogen receptor + (%)	494 (73.0%)	/
HER2 + (%)	347 (51.3%)	/
Ki_67 ≥ 14% (%)	584 (86.3%)	/
CK5_6 + (%)	101 (15.0%)	/
EGFR + (%)	135 (20.0%)	/
**Metastasis**		
Number of patients with liver metastasis (%)	21 (3.1%)	/
Number of patients with lung metastasis (%)	21 (3.1%)	/
Number of patients with lymph metastasis (%)	42 (6.2%)	/
CEA (median [IQR])	1.82 [1.22, 2.77]	1.36 [0.88, 1.95]
CA125 (median [IQR])	14.90 [9.90, 23.60]	15.10 [10.10, 20.45]
CA153 (median [IQR])	13.60 [7.90, 21.00]	8.85 [6.03, 14.60]
HSP90AA1 (median [IQR])	64.80 [40.35, 93.10]	41.40 [29.38, 71.85]
Tcell (median [IQR])	67.40 [61.60, 73.00]	67.55 [59.75, 73.95]
Th (median [IQR])	39.50 [34.27, 44.52]	38.09 [31.83, 42.76]
Tc (median [IQR])	20.70 [16.70, 25.20]	20.70 [15.70, 25.60]
NK (median [IQR])	12.00 [8.47, 17.20]	10.24 [5.95, 14.60]
Bcell (median [IQR])	12.73 [9.70, 16.47]	11.60 [8.42, 15.59]
NEUT (median [IQR])	3.61 [2.87, 4.65]	3.85 [3.05, 4.73]
LYMPH (median [IQR])	1.77 [1.43, 2.13]	1.79 [1.44, 2.10]
MONO (median [IQR])	0.37 [0.29, 0.45]	0.34 [0.28, 0.42]
PLT (median [IQR])	258.00 [220.00, 305.00]	262.50 [217.00, 297.75]
D-dimer (median [IQR])	0.16 [0.07, 0.36]	0.12 [0.04, 0.22]

*EGFR, epidermal growth factor receptor; CEA, carcinoembryonic antigen, ng/mL; CA125, carbohydrate antigen 125, ng/mL; CA153, carbohydrate antigen 153, ng/mL; HSP90AA1, heat shock protein 90-alpha, ng/mL; Th, helper T cell,%; Tc, killer T cell,%; NK, natural killer,%; NEUT, neutrophile, 10^–9^/L; LYMPH, lymphocyte, 10^–9^*/*L; MONO, monocyte, 10^–9^/L; PLT, platelet, 10^–9^/L; D-dimer, mg/L; SD, standard deviation; IQR, interquartile range.*

**FIGURE 5:**
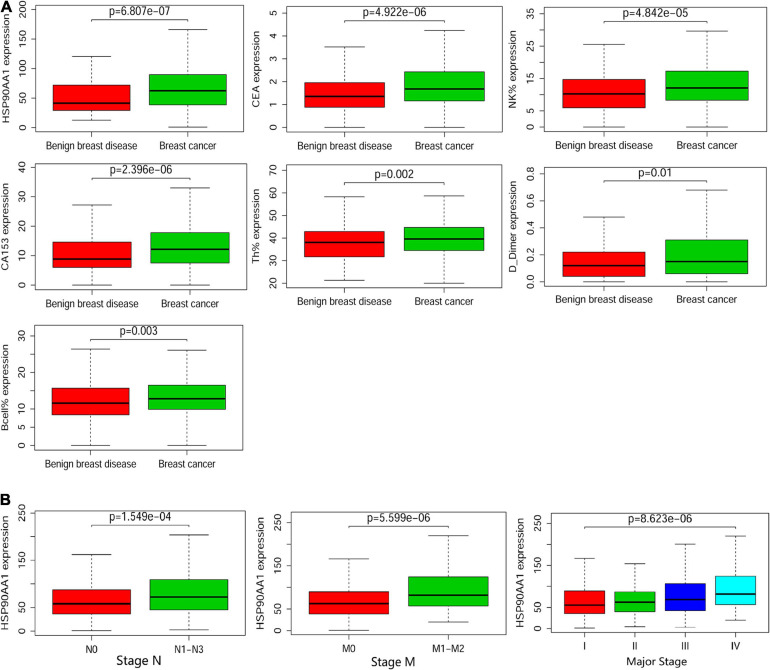
Boxplots of the cancer and metastasis risks according to the cohort. **(A)** Boxplots of the significantly elevated clinical indicators in benign breast disease and in stage I, II, and III breast cancer. **(B)** Boxplots of HSP90α according to clinical significance in breast cancer patients. Differences between groups were estimated using the Mann–Whitney U or Kruskal–Wallis test, as appropriate.

The levels of different detection indicators in the plasma were compared between patients with breast cancer and benign breast disease (Supplementary Figure 3A). HSP90AA1 was significantly elevated in breast cancer patients (Figure 5A; *p* = 6.807e-07; Wilcoxon test). Moreover, HSP90AA1 expression was significantly different between patients in different subgroups sorted by clinical stage (*p* = 8.623e-06, Kruskal–Wallis test), histological N grade (*p* = 1.549e-04, Wilcoxon test), and histological M grade (*p* = 5.599e-06, Wilcoxon test) (Figure 5B). However, HSP90AA1 expression showed no difference among other clinical groups (Supplementary Figure 3B).

### Receiver Operating Characteristic (ROC) Curves

**FIGURE 6:**
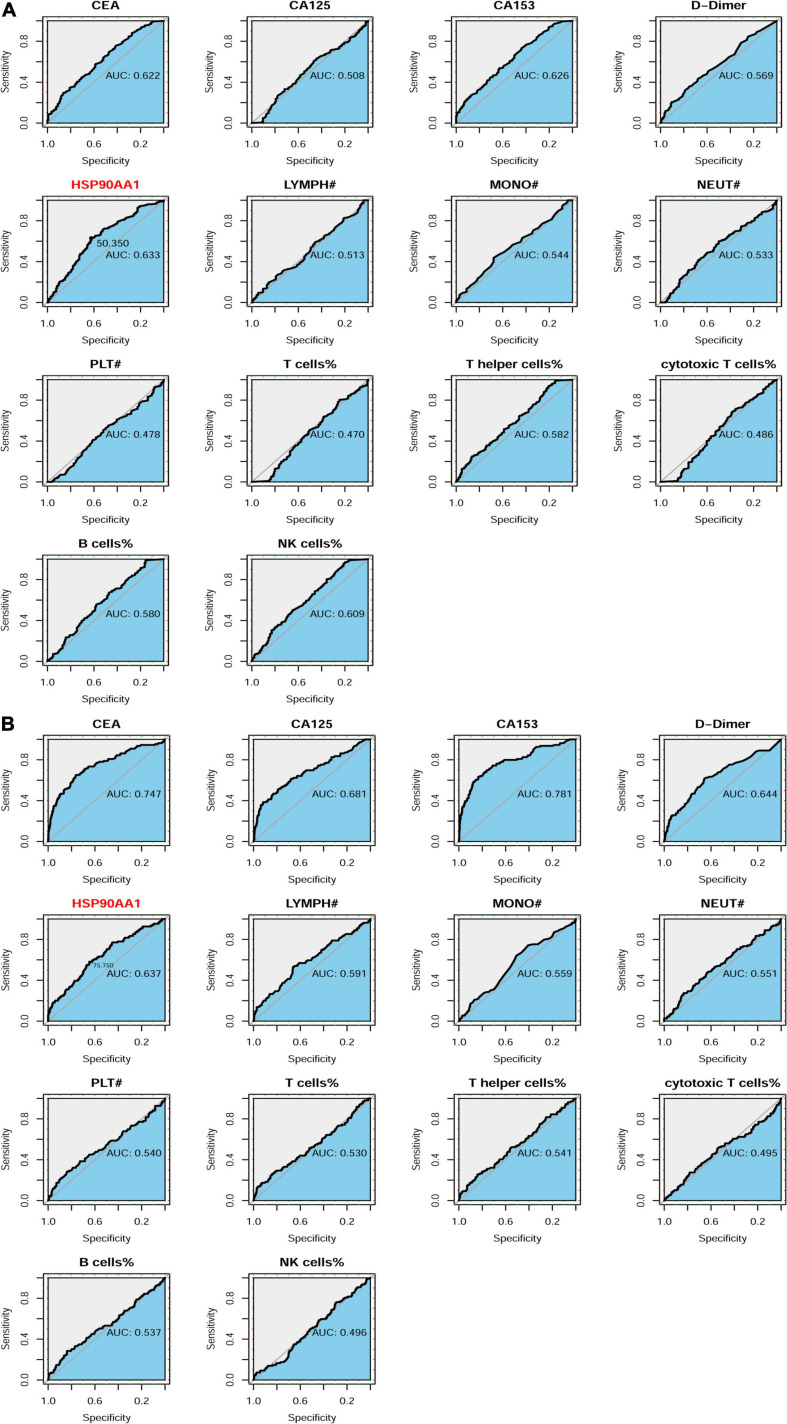
Patient enrollment in this study and ROC curve analyses. **(A)** Receiver operating characteristic (ROC) curve analysis to evaluate the predictive value of each marker for definite tumor diagnosis. HSP90AA1 shows the highest accuracy for the prediction of breast cancer compared with that of other markers. **(B)** ROC curve analysis to evaluate the predictive value of each marker for the definite diagnosis of breast cancer metastasis.

We compared the area under the curve (AUC) values between all of the detection indicators in the two cohorts. The AUC value of HSP90AA1 was significantly higher than that of other markers in the cancer risk cohort. Next, we used ROC curve analysis (indexed by Youden’s index) to determine the optimal cut-off value for the cancer risk cohort (HSP90AA1: 50.35, Figure 6A). Next, we investigated the significance of HSP90AA1 levels in the metastasis of breast cancer. ROC curves were drawn to analyze the optimal cut-off values, diagnostic sensitivities, and specificities (HSP90AA1: 75.75, Figure 6B).

### Construction of the Cancer Risk and Metastasis Risk Nomograms Containing HSP90AA1

**FIGURE 7:**
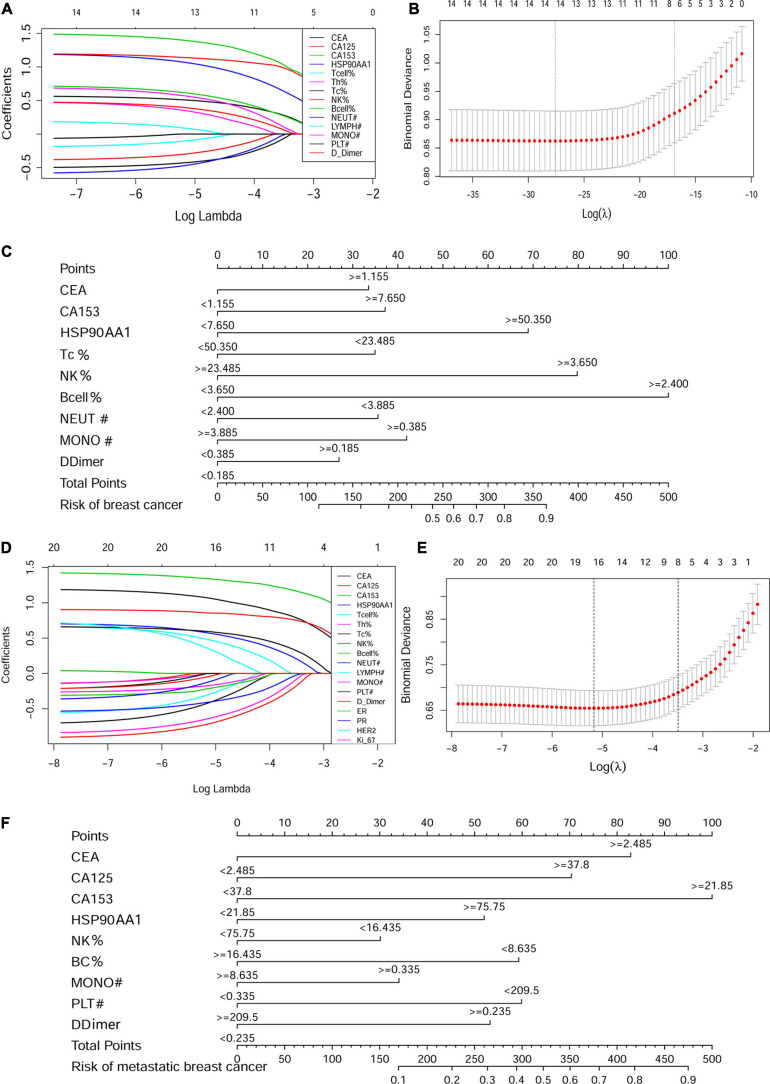
Construction of the cancer risk and metastasis risk nomograms containing HSP90AA1. LASSO coefficient profiles of the **(A)** 14 factors in the cancer risk cohort and **(D)** 21 factors in the metastasis risk cohort. A coefficient profile plot was produced against the logλ sequence. **(B,E)** Selection results of the LASSO model. The partial likelihood deviance (binomial deviance) curve is plotted versus logλ. Dotted vertical lines are drawn at the optimal values by using the minimum criteria and the 1 SE of the minimum standards (the 1-SE rules). Development of the **(C)** breast cancer risk nomogram and **(F)** metastasis risk nomogram. LASSO, least absolute shrinkage and selection operator.

Of the cancer risk cohort, 14 functional markers were all included and had non-zero coefficients in the LASSO regression model (Figures 7A,B). Nomograms were constructed based on the logistic regression analysis among these independent predictors (Supplementary Figure 4A). Based on the nomograms, HSP90AA1, B cells, and NK cells contribute the most to diagnosis, followed by CEA, CA153, cytotoxic T cells, neutrophils, monocytes, and d-dimers (Figure 7C).

Next, in the metastasis risk cohort, 21 clinical-pathological features and biomarkers in the peripheral blood were reduced to 14 possible predictors, which had non-zero coefficients in the LASSO regression model (Figures 7D,E). The results of the logistic regression analysis of these independent predictors are shown in Supplementary Figure 4B. The predicted model, which contained CEA, CA125, CA153, HSP90AA1, NK cells, B cells, monocytes, platelets, and d-dimers, was established and presented in the form of a nomogram (Figure 7F).

### Calibration and Validation

**FIGURE 8:**
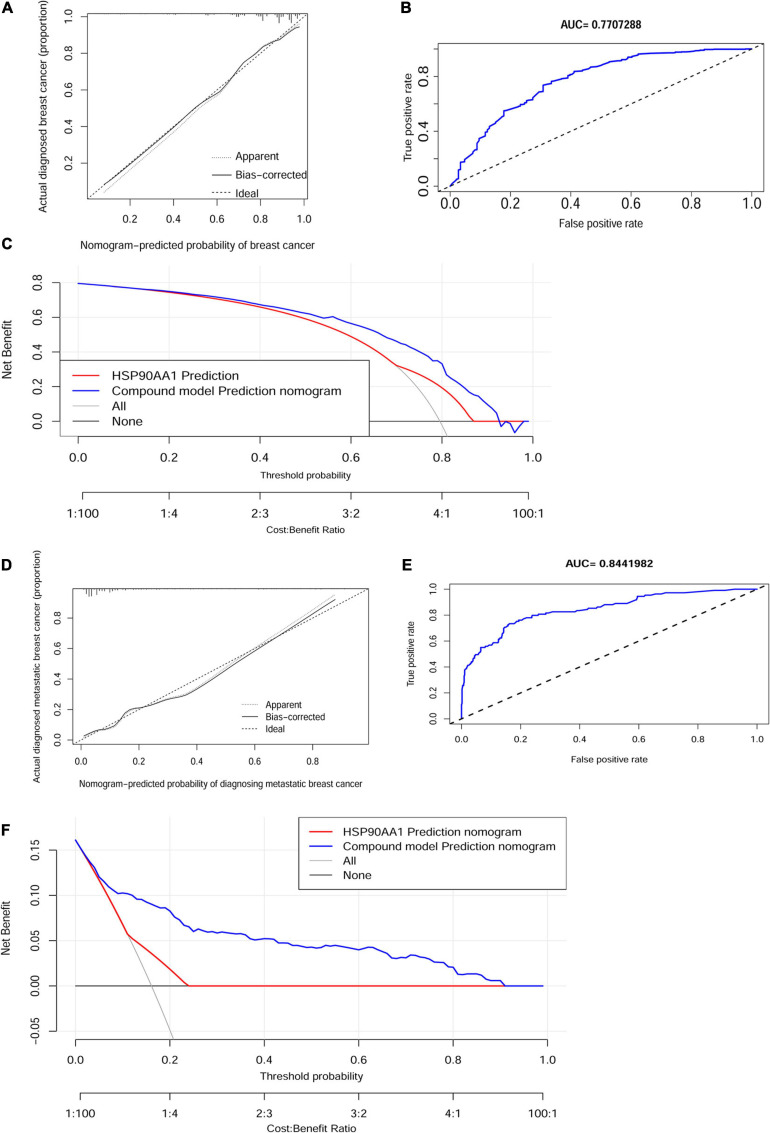
Calibration and validation. Calibration curves of the non-adherence nomogram prediction for **(A)** cancer risk and **(D)** metastasis risk. The *x*-axis represents the predicted risk whereas the *y*-axis represents the diagnosed value. The diagonal dotted line represents perfect prediction by an ideal model. The solid line represents the performance of the nomogram, of which a closer fit to the diagonal dotted line represents better prediction. Receiver operating characteristic curve of **(B)** the cancer risk model and **(E)** metastasis risk. Decision curve analysis for **(C)** the breast cancer risk nomogram and **(F)** the metastasis risk nomogram. The *X*-axis is the risk threshold probability that changes from 0 to 1, and the *Y*-axis is the calculated net benefit for a given threshold probability. AUC, area under the curve.

The calibration curve of the cancer risk nomogram and metastatic risk nomogram for the prediction of cancer risk and metastasis risk in females demonstrated significant agreement in these cohorts (Figures 8A,D). The AUCs were 77.1% [95% CI: 72.5–81.7%] and 84.4% [95% CI: 80.1–88.7%], which were higher than that of the AUC of any single factor (Figures 8B,E). The C-indexes for the prediction nomogram were 0.77 and 0.84, which were confirmed to be 0.75 and 0.83 through bootstrapping validation. Decision curve analysis and clinical impact plot were used to determine the clinical utility of risk prediction nomograms. The decision curve analysis showed that if patients and doctors’ threshold probabilities are 5–92% and 1 –90%, respectively, this progression nomogram could add more benefit than the scheme to predict the risk of onset and metastasis (Figures 8C,F). The results indicated that HSP90AA1 was not a decisive factor in diagnosing metastasis in breast cancer; however, it improved diagnostic accuracy. The clinical impact plot provided corresponding verification (Supplementary Figure 5).

### Metastasis Risk Nomogram in Patients With Different Molecular Types of Breast Cancer

**FIGURE 9:**
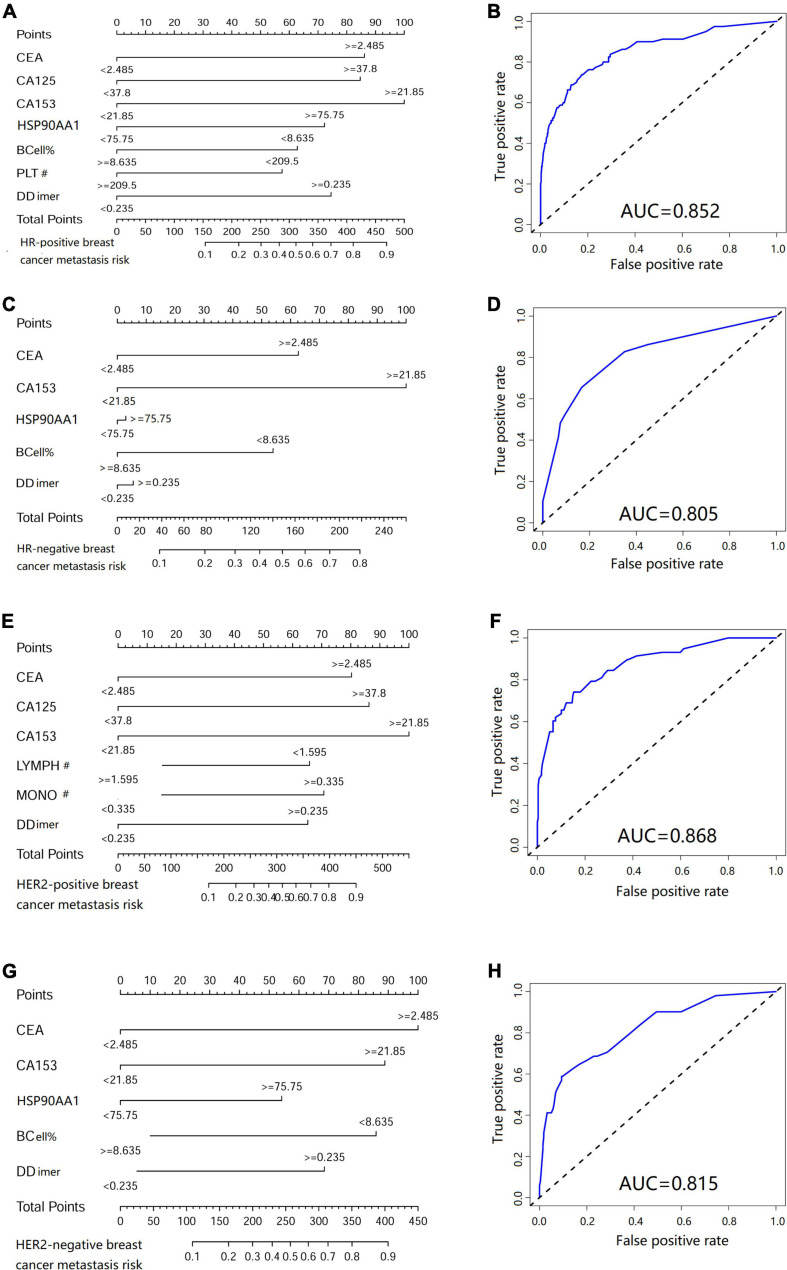
Metastasis risk nomogram in patients with different molecular types of breast cancer. Metastasis risk nomograms of **(A)** hormone receptor (HR) positive and **(C)** negative patients. Metastasis risk nomograms of **(E)** human epidermal growth factor receptor-2 (HER2) positive and **(G)** negative patients. Receiver operating characteristic curve of HR **(B)** positive, **(D)** negative, HER2 **(F)** positive and **(H)** negative patients.

In HR negative/positive and HER2 negative/positive patients, 21 clinicopathological characteristics and biomarkers in peripheral blood were screened and a nomogram for metastasis prediction was established (Table 1 for the number of patients in each group). We found that the nomograms of patients with different molecular types are different. Among them, the predictive nomogram of HR positive/negative patients is consistent with the metastasis cohort (Figure 9A), but the predictive ability of HR negative patients is reduced (Figure 9C). The metastasis prediction model for HER2-negative patients includes CEA, CA153, HSP90AA1, B cells, and d-dimers (Figure 9E). HSP90AA1 was not included in the metastasis prediction model for HER2-positive patients (Figure 9G). But the AUCs of the four models are all greater than 80% (Figures 9B,D,F,H). The model calibration and verification results are shown in Supplementary Figure 6.

### Western Blotting Methods Verification Levels of Plasma Hsp90AA1 From Partial ELSIA Negative Patients

Finally, Because Wei’s reported the uncertainty of the diagnosis of plasma Hsp90AA1 and the heterogeneity of the HSP90AA1 protein secreted by tumor cells, we randomly screened the plasma of 32 ELISA-negative breast cancer patients. We used the polyclonal antibodies with clear characteristics for western blotting. It was found that in some ELISA-negative breast cancer patients, the secretion level of plasma Hsp90AA1 detected by western blotting was higher than the critical value (Supplementary Figure 7A). Still, the overall results were consistent with the ELISA test results (Supplementary Figure 7B).

## Discussion

In this study, we investigated the expression patterns and prognostic values of different HSP90 family members (*HSP90AA1*, *HSP90AA2*, *HSP90AB1*, *HSP90B1*, and *TRAP1*) in breast cancer. Further, we included 677 patients with breast cancer and 146 with breast benign disease who underwent surgery and medical treatment. These findings may provide the means to improve the accuracy of the diagnosis and prognosis of breast cancer patients. The primary role of heat shock proteins (HSPs) in tumorigenesis is to stabilize abnormally expressed tumor-related genes (Calderwood and Gong, 2016). HSPs are released from cancer cells and affect their properties and functions through receptor-mediated signal transduction (Ono et al., 2018). Previous reports have shown that HSPs are overexpressed in melanoma and colon cancer and are related to the excessive activation of the Wnt signaling pathway (Skrzypczak et al., 2010). HSP90 is important for the stabilization and activation of more than 200 proteins, many of which are essential for cell signaling and adaptive responses to stress (Schopf et al., 2017). HSP90 forms a dynamic complex of HSP90 chaperons with other HSPs (Young et al., 2003). Therefore, HSP90 is considered an essential promoter of cancer cell survival, especially in breast cancer (Whitesell and Lindquist, 2005; Vartholomaiou et al., 2017). In our study, we confirmed that the expression of HSP90s in breast cancer tissues was significantly higher than that in normal tissues. We also observed a significant correlation between the expression of *HSP90AA1* and tumor stage in breast cancer patients. The OS of breast cancer patients with high *HSP90AA1* expression was low.

Accumulating studies have demonstrated that genes within the HSP90 family are potentially involved in the pathogenesis of human malignancies. Although previous research has shown that they can predict tumor development, the expression levels of HSP90s are rarely used as tumor diagnostic biomarkers (Trepel et al., 2010). Some studies believe that some HSP90 family genes such as *HSP90AA1* and *TRAP1* are not necessary for breast tumors to develop and metastasize; however, they have surprising regulatory effects (Vartholomaiou et al., 2017). *HSP90AA2* gene polymorphism has been reported to be related to certain immune diseases (Zhang et al., 2018), but there is no report related to tumors. *HSP90AB1* is a closed homolog of *HSP90AA1*, which is necessary for large-scale cellular processes and, therefore, is essential for cell survival (Haase and Fitze, 2016). Studies have suggested that *HSP90AA1*, *HSP90AB1* gene products, and their associated chaperon proteins (Aha1, Cdc37, p23, and Tpr2) as well as HSP90-dependent transcription factor HSF1 are overexpressed in a variety of cancers (McDowell et al., 2009). High mRNA expression driven by amplification of the chromosome coding region of *HPS90AB1* was found to be associated with poor prognosis of HER2-negative/ER-positive breast cancer (Cheng et al., 2012). In our study, the ONCOMINE and TCGA data sets showed that the expression of *HPS90AB1* in breast cancer tissues was higher than that in healthy tissues, but the difference was not significant. The high expression of *HPS90AB1* was closely related to the overall poor survival in breast cancer patients who were followed up with for more than 250 months. *HSP90B1* is an HSP90 paralog found in the endoplasmic reticulum; it plays critical roles in folding proteins in the secretory pathway, such as Toll-like receptors and integrins (Randow and Seed, 2001). Dejean and Liu showed that the expression level of *HSP90B1* in recurrent human breast cancer was higher than that of its matched primary tumor. Further, it was found to be an independent and unfavorable prognostic indicator of breast cancer survival (Dejeans et al., 2012; Liu et al., 2018). Our research suggested that the imbalance of *HSP90B1* was not closely related to the occurrence or development of breast cancer, and had no effect on the prognosis of breast cancer. In summary, our results indicated that although gene expression levels of *HSP90AA1*, *HSP90AA2*, *HSP90AB1*, *HSP90B1*, and *TRAP1* were upregulated in breast cancer patients, only the expression of *HSP90AA1* was related to the tumor stage and prognostic survival.

Based on the above findings, it is expected that *HSP90AA1* could act as a potential diagnostic and prognostic biomarker for breast cancer. According to the definition of the term “biomarker” by the National Institutes of Health Biomarker Definition Working Group (Boers et al., 2014), HSP90AA1 is currently recognized as a signature that is independent of conventional classifications (Fu et al., 2017). In our research, we did not find that HSP90AA1 expression was highly correlated with other detection indicators; HSP90AA1 is expected to predict a patient’s response or adverse effect to a specific treatment by improving their prognosis and quality of life (Boers et al., 2014). HSP90AA1 is inexpensive, readily available, simplified, and used objective approaches to inform clinical decision-making and stratify patients into different risk groups (Boers et al., 2014; Carethers and Jung, 2015; Fu et al., 2017; Okugawa et al., 2020). In this study, the expression of HSP90AA1 was significantly different between tumors and precancerous lesions. Further analysis of tumor patients revealed that HSP90AA1 was highly correlated with N and M stages, showing its distinguishing power for metastatic patients. Subsequently, we used the ROC curve analysis to adopt two different cut-off points for the risk of disease and the risk of metastasis. Compared with the traditional medical reference values, they showed higher accuracy despite the established evidence for B-cell, CEA, and CA153 for assessing the risk of recurrence and prognosis in breast cancer patients (Coronella-Wood and Hersh, 2003; Dai et al., 2016). Using established biomarkers for breast cancer which are currently available in clinical practice, we first developed a predictive model including HSP90AA1, which can be used to calculate breast cancer risk for breast disease and risk of distant metastasis in breast cancer. We explored the predictive value of pre-therapeutic plasma HSP90AA1 levels and developed nomograms that include HSP90AA1. These novel nomograms may help to facilitate breast cancer risk prediction and metastasis risk prediction in breast cancer patients.

The strengths of this study include its broadly clinically applicable prospects. The protein levels of HSP90AA1 were significantly elevated in tumors compared to normal mammary glands (Vartholomaiou et al., 2017). This was also observed in a breast cancer diagnostic study; they used fluorescein HS-27 in combination with HSP90 to provide a cost-effective and easy-to-implement diagnostic platform (Crouch et al., 2019). The biochemical assessment may lead to savings of almost 50% compared with medical imaging techniques (Robertson et al., 1995). Therefore, we developed and validated a novel prediction tool for breast cancer risk using a few readily available variables. Incorporating tumor markers, inflammation markers, and cell-mediated immunity markers into an easy-to-use nomogram can facilitate the prediction of risk. This study provided a relatively accurate prediction tool. Internal validation in the cohort demonstrated good discrimination and calibration power; in particular, our high C-indexes (0.77 and 0.84) for interval validation identified that this nomogram could be widely used (Wei et al., 2017). Seven indicators including HSP90, CEA, CA153, NK, B-cell, monocyte count, and d-dimer were incorporated into both models. NK, B-cell, monocyte count and d-dimer have also been reported to be related to tumor metastasis in previous studies (Liu et al., 2016; Tang et al., 2020). To the best of our knowledge, this study is the first to combine nomograms with commonly used tumor markers and HSP90 for breast cancer risk assessment and metastasis. Through this convenient evaluation method, both clinicians and high-risk patients can quickly obtain personalized risk predictions. Further, this method may be beneficial in estimating high-risk groups and guiding follow-up treatment. Although it has been reported that the expression of HSP90AA1 in tissues is significantly correlated with HR and HER2 (Klimczak et al., 2019), the expression of free HSP90AA1 is not significantly different in patients with HR (ER, PR) and HER2 positive/negative subgroups (Supplementary Figure 3B). Furthermore, in the HER2-positive subgroup, free HSP90AA1 is not a risk factor for distant metastasis in this group than other HR and HER2-negative subgroups.

Our retrospective study also has some limitations. First, our study relied exclusively on a single institutional database, although eligibility criteria were formulated to minimize selection bias. Second, the accuracy of our nomograms should be assessed via external validation, which would help to evaluate whether our nomograms are appropriate for a new population; if so, they could be generalized to other people. Further clinical trials (including a prospective cohort study) are required to illustrate and improve the validity of this model for therapeutic decision-making for breast cancer. In addition, for the experimental method we use, the ELISA kit only quantifies the target protein through the exposed epitopes in the solution instead of detecting the total protein. The interaction between the target antigen and antibody in the kit can also be affected by autoantibodies, other binding proteins, and even post-translational modification. These will lead to a decline in the diagnostic performance of the current ELISA kit (Liu et al., 2019), which will affect the application value of the model in this article. In the later stage, we will use Western blot and proteomics methods to improve our results.

## Conclusion

In conclusion, our systematic and comprehensive analysis determined that HSP90AA1 of the HSP90 family can be a good diagnostic marker for breast cancer disease and metastasis. HSP90AA1 is a new disease and metastasis risk evaluation index for breast cancer patients is essential. Our study revealed that appropriate use of pretreatment plasma HSP90AA1 levels in combination with other markers could more effectively predict the patient’s cancer and metastasis rates. Quantification of preoperative HSP90AA1 may help physicians to more effectively manage risk and to determine optimal postoperative oncological follow-up strategies for patients with breast cancer.

## Data Availability Statement

The datasets presented in this study can be found in online repositories. The names of the repository/repositories and accession number(s) can be found below: https://figshare.com/articles/figure/Plasma_HSP90AA1_Predicts_the_Risk_of_Breast_Cancer_Onset_and_Distant_Metastasis/14349362.

## Ethics Statement

The studies involving human participants were reviewed and approved by the Ethics Committee of Guangxi Medical University Cancer Hospital (LW2020065). The patients/participants provided their written informed consent to participate in this study.

## Author Contributions

HL, KL, and LZ designed the study. ZZ, YH, JL, and WW performed the experiments. HL and SN analyzed the data. HL wrote the manuscript. All authors approved the final version of the manuscript.

## Conflict of Interest

The authors declare that the research was conducted in the absence of any commercial or financial relationships that could be construed as a potential conflict of interest.

## Footnotes

1 https://www.oncomine.org

2 http://gepia2.cancer-pku.cn

3 http://ualcan.path.uab.edu/index.html

4 https://www.proteinatlas.org

## Abbreviations

AUC: area under the curve
B cell: B lymphocytes%
CA125: carbohydrate antigen 125
CA153: carbohydrate antigen 153
CEA: carcinoembryonic antigen
CI: confidence interval
C-index: concordance index
D_Dimer: d-dimer
EGFR: epidermal growth factor receptor
ER: estrogen receptor
GEPIA: Gene Expression Profiling Interactive Analysis
HER2: human epidermal growth factor receptor 2
HSP90s: HSP90 family genes
LASSO: least absolute shrinkage and selection operator
LYMPH#: lymphocyte count
MONO#: monocyte count
NEUT#: neutrophil count
NK cell: natural killer lymphocytes%
OS: overall survival
PLT#: platelet count
PR: progesterone receptor
ROC: receiver operating characteristic
T cell: T lymphocytes%
Tc: cytotoxic T cells%
TCGA: the Cancer Genome Atlas
Th: helper T cell%
THPA: the Human Protein Atlas.
