# Supplementary material for: Plasma HSP90AA1 Predicts the Risk of Breast Cancer Onset and Distant Metastasis
Source: Front Cell Dev Biol. 2021 May 24;9:639596. doi: 10.3389/fcell.2021.639596 (PMC8181396; doi:10.3389/fcell.2021.639596)
Supplement: Supplementary file 1 [file Image_1.pdf]

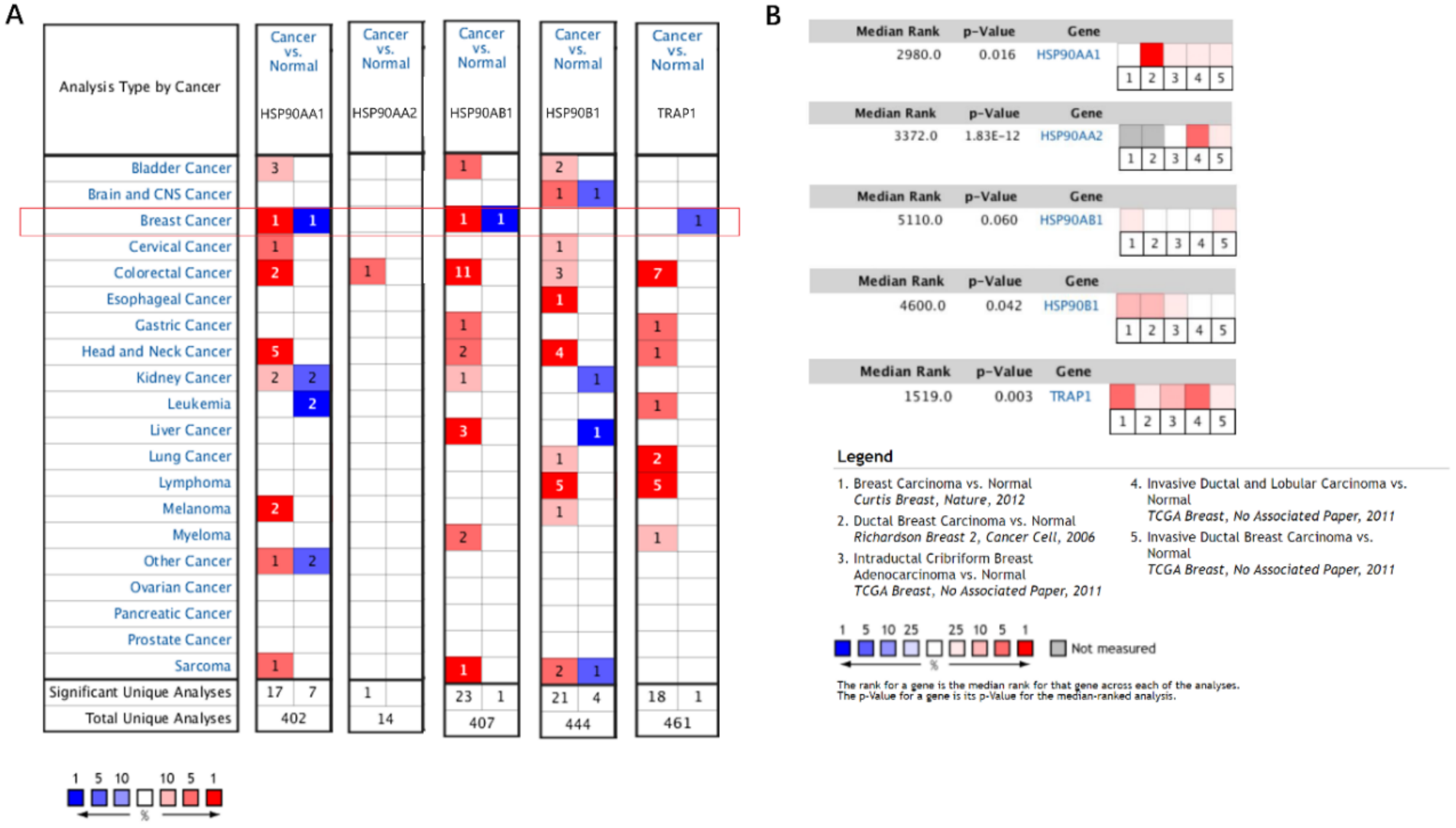

Supplementary Figure 1. Heat shock protein 90 kDa family gene analysis in breast cancer (Oncomine database). (A) The number of up-regulated datasets was found to be greater than the number of down-regulation datasets for most HSP90s, except for HSP90AA2, in various types of tumors compared with normal tissues. Red: over-expression; blue: down-regulation. (B) HSP90AA1, HSP90AA2, HSP90AB1, HSP90B1, and TRAP1 were significantly upregulated in five datasets, including TCGA.
