# Supplementary material for: Plasma HSP90AA1 Predicts the Risk of Breast Cancer Onset and Distant Metastasis
Source: Front Cell Dev Biol. 2021 May 24;9:639596. doi: 10.3389/fcell.2021.639596 (PMC8181396; doi:10.3389/fcell.2021.639596)
Supplement: Supplementary file 2 [file Image_2.pdf]

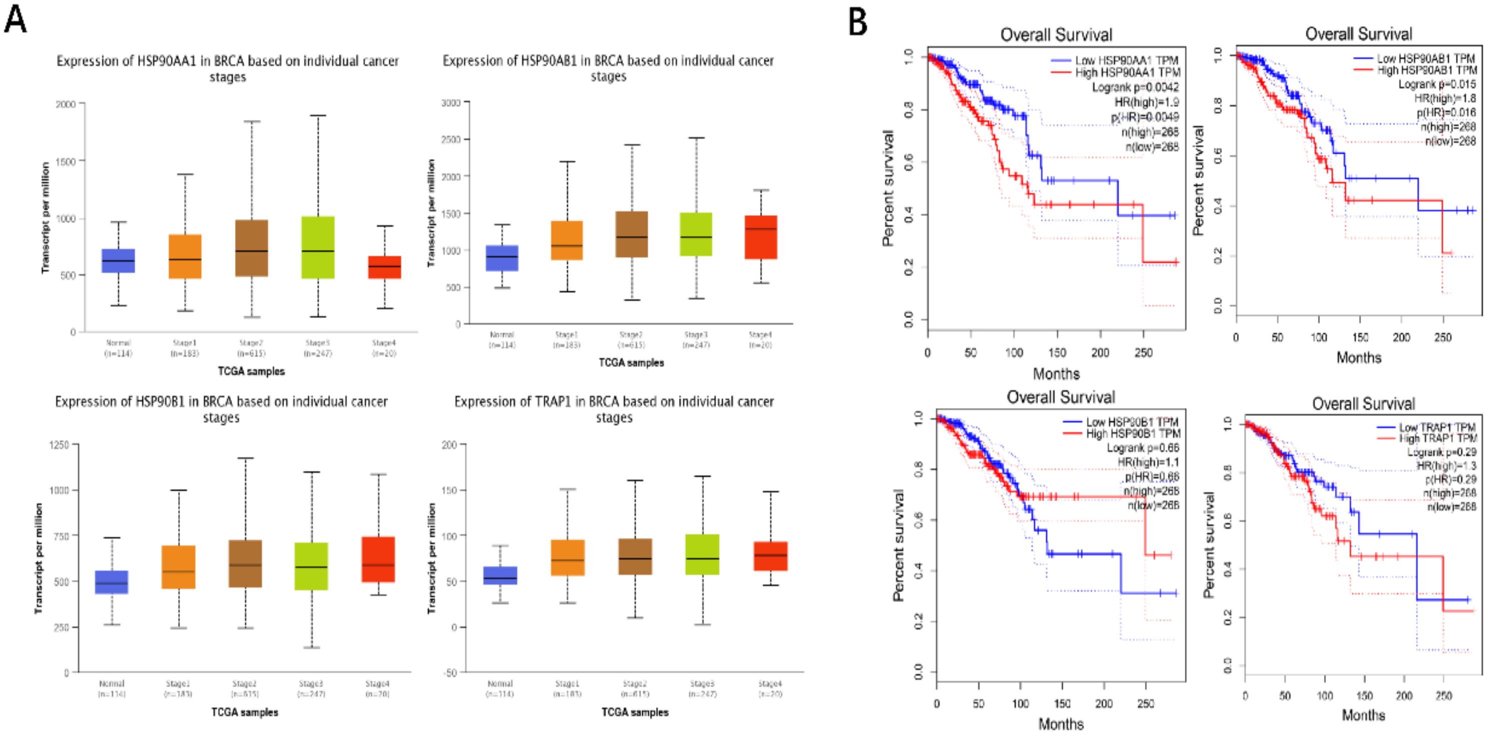

Supplementary Figure 2. Correlations of the expression levels of HSP90s with tumor stages and prognostic values of HSP90s in breast cancer patients. (A) The UALCAN is used to determine the relationship between the expression levels of *HSP90AA1, HSP90AB1, HSP90B1*, and *TRAP1* and tumor stages in breast cancer patients ($p < 0.05$). The x-axis indicates the stage of breast cancer tumors, and the y-axis shows the expression level of HSP90s (gene expression ~ pathological stage). (B) The overall survival curves comparing breast cancer patients with high (red) and low (blue) HSP90s expression levels are plotted using the GEPIA**.**
