# Supplementary material for: Plasma HSP90AA1 Predicts the Risk of Breast Cancer Onset and Distant Metastasis
Source: Front Cell Dev Biol. 2021 May 24;9:639596. doi: 10.3389/fcell.2021.639596 (PMC8181396; doi:10.3389/fcell.2021.639596)
Supplement: Supplementary file 3 [file Image_3.pdf]

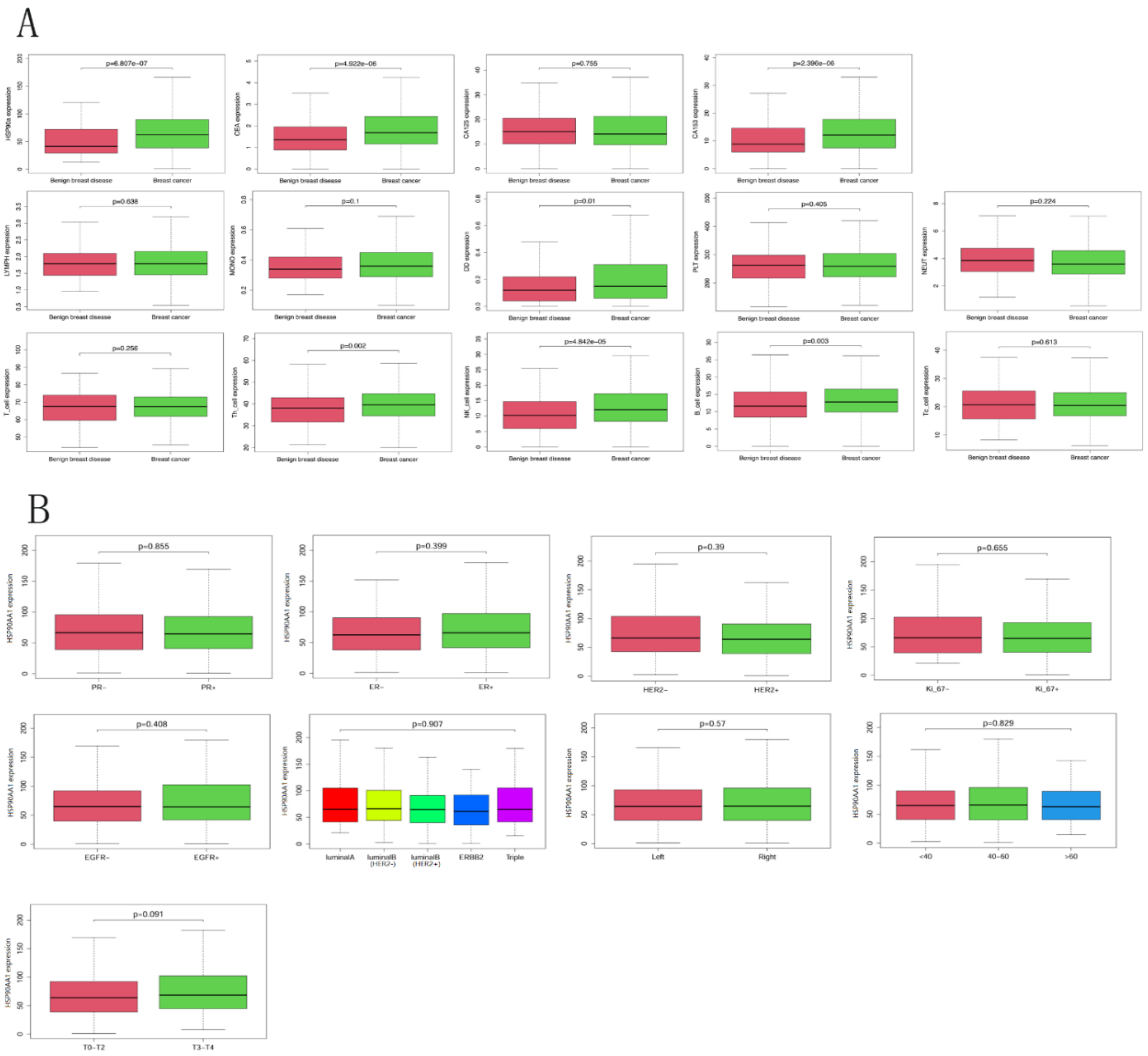

Supplementary Figure 3. Boxplots of the cancer risk and metastasis risk for the different cohorts. (A) Boxplots of clinical indicators in benign breast disease and in stage I, II, and III breast cancer. (B) Boxplots of HSP90AA1in different breast cancer patients. The clinical indicators analyzed using immunohistochemical analysis include: ER, PR, HER2, Ki-67, CK5/6, and EGFR; molecular types of breast cancer (luminal A, luminal B(HER2-), luminal B(HER2+), ERBB2, triple; the left and right breasts; and age. Differences between groups were estimated using Mann–Whitney U or Kruskal–Wallis tests, as appropriate.
