# Supplementary material for: Plasma HSP90AA1 Predicts the Risk of Breast Cancer Onset and Distant Metastasis
Source: Front Cell Dev Biol. 2021 May 24;9:639596. doi: 10.3389/fcell.2021.639596 (PMC8181396; doi:10.3389/fcell.2021.639596)
Supplement: Supplementary file 4 [file Image_4.pdf]

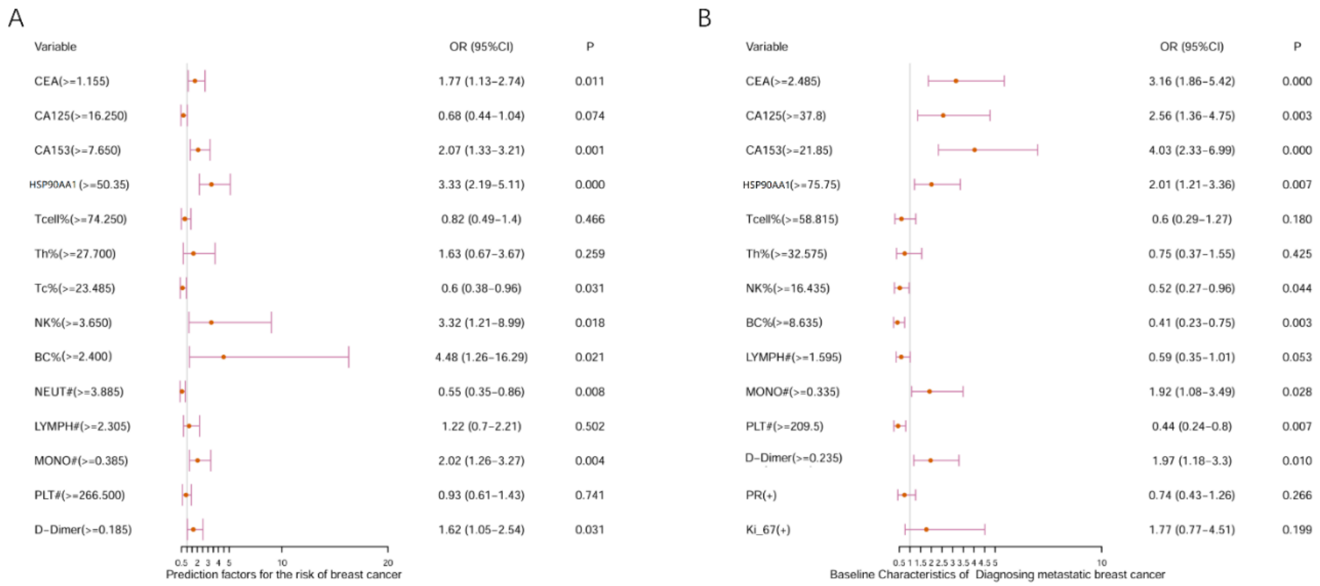

Supplementary Figure 4. Prediction factors for our research. (A) Cancer risk cohort, (B) The metastasis risk cohort.
