# Supplementary material for: Plasma HSP90AA1 Predicts the Risk of Breast Cancer Onset and Distant Metastasis
Source: Front Cell Dev Biol. 2021 May 24;9:639596. doi: 10.3389/fcell.2021.639596 (PMC8181396; doi:10.3389/fcell.2021.639596)
Supplement: Supplementary file 5 [file Image_5.pdf]

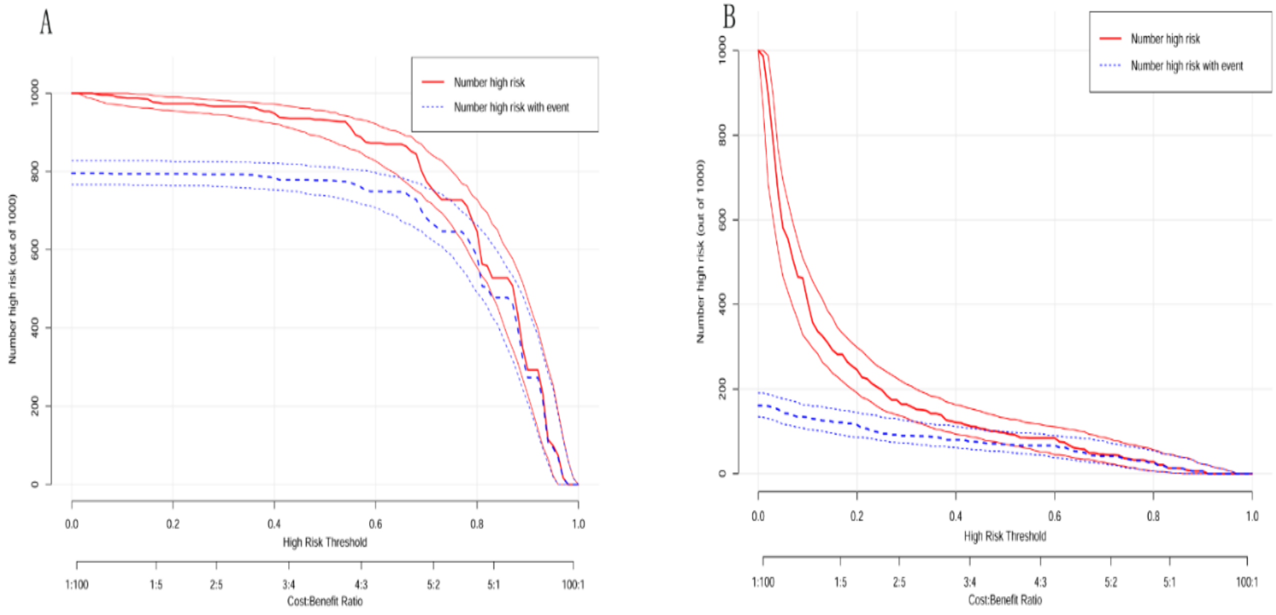

Supplementary Figure 5. Clinical impact plots of the risk prediction model. Of 1000 patients, the red curve represents the number of people who are classified as positive (high risk) by the simple model at each threshold probability, The blue curve shows how many of those would be true positives (cases). (A) Cancer risk cohort; (B) the metastasis risk cohort.
