# Supplementary material for: Plasma HSP90AA1 Predicts the Risk of Breast Cancer Onset and Distant Metastasis
Source: Front Cell Dev Biol. 2021 May 24;9:639596. doi: 10.3389/fcell.2021.639596 (PMC8181396; doi:10.3389/fcell.2021.639596)
Supplement: Supplementary file 6 [file Image_6.pdf]

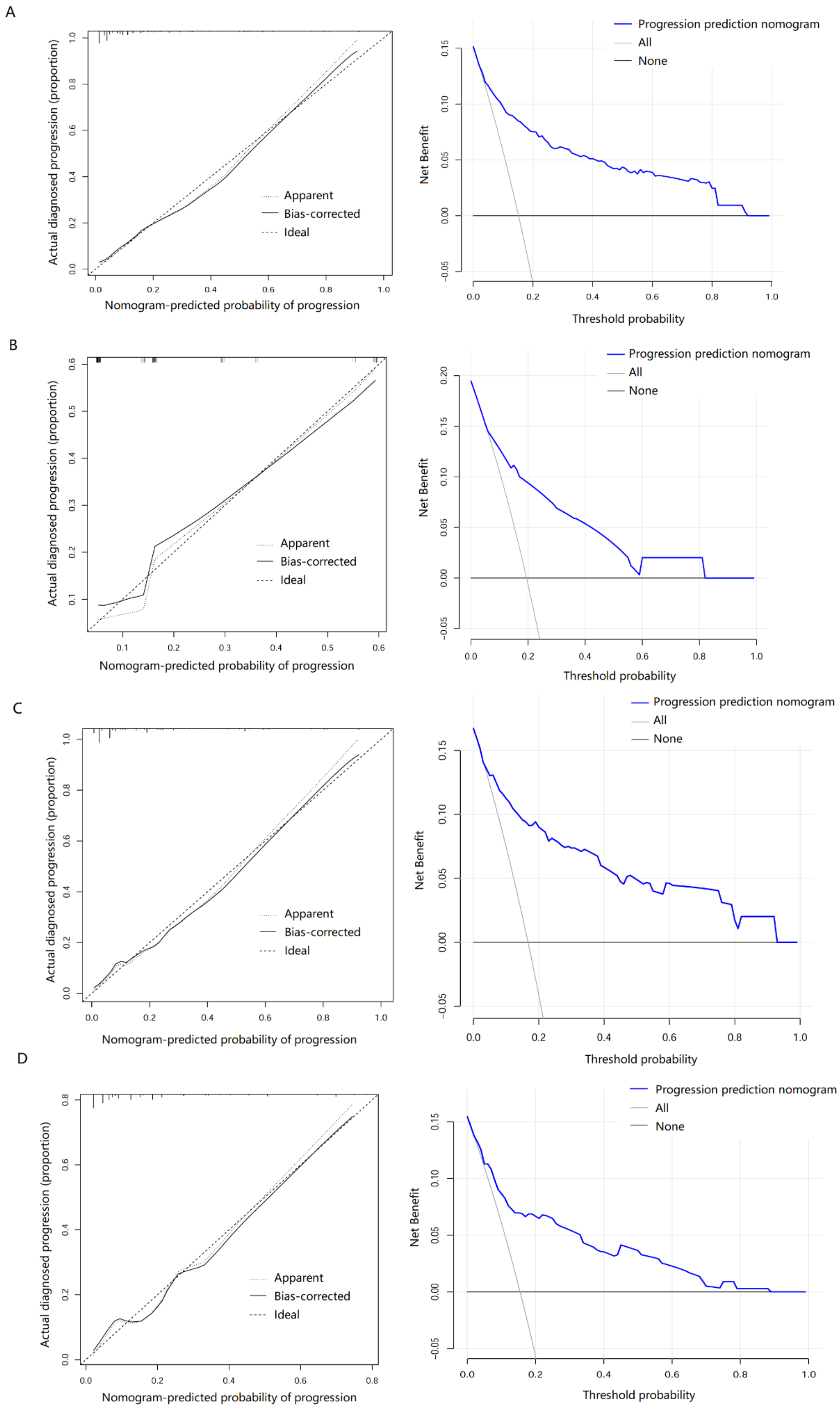

Supplementary Figure 6. Calibration curves of the nomogram prediction in patients with different molecular types metastasis risk. HR (A) positive, (B) negative, HER2 (C) positive, and (D) negative patients**.**
