# Supplementary material for: Plasma HSP90AA1 Predicts the Risk of Breast Cancer Onset and Distant Metastasis
Source: Front Cell Dev Biol. 2021 May 24;9:639596. doi: 10.3389/fcell.2021.639596 (PMC8181396; doi:10.3389/fcell.2021.639596)
Supplement: Supplementary file 7 [file Image_7.pdf]

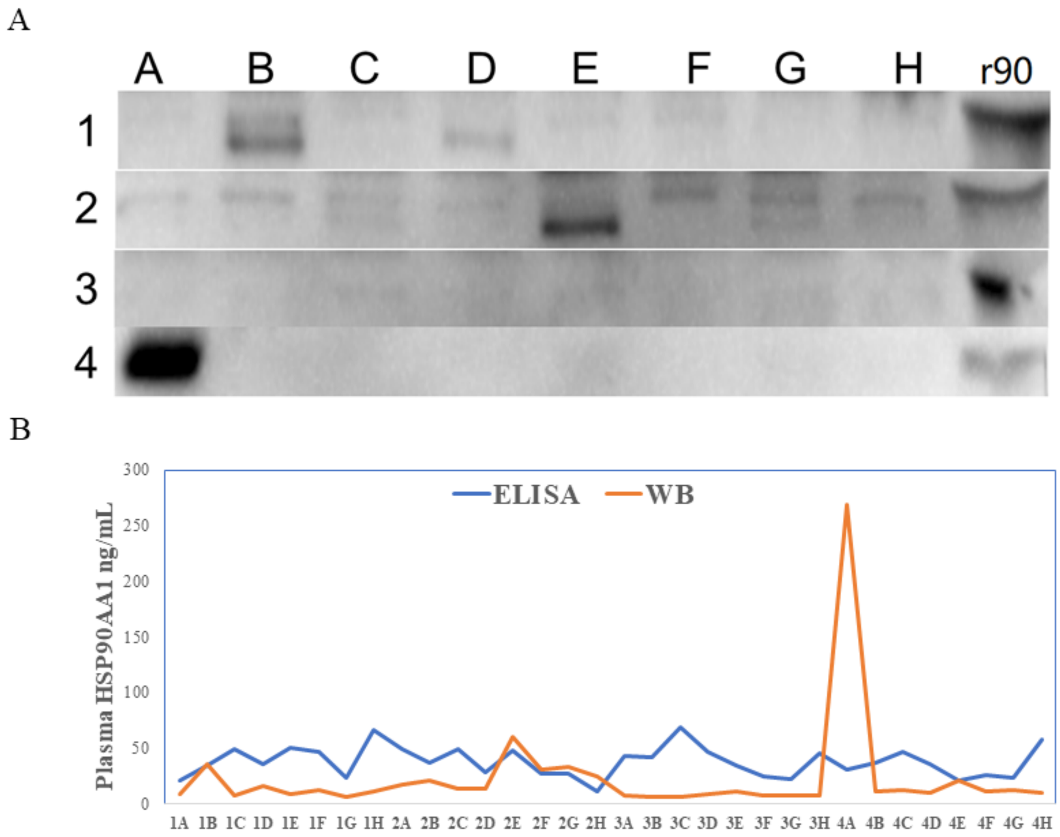

Supplementary Figure 7. Western blotting (WB) methods verification levels of plasma Hsp90AA1 from partial ELSIA negative patients. (A) Detect plasma Hsp90AA1 in ELISA negative breast cancer patients by Western blotting. r90, recombinant Hsp90AA1 (75 ng/mL). (B) Plasma Hsp90AA1 concentration profiles categorized in ELISA negative patients with breast cancer: ELISA and WB.
