# Supplementary material for: Plasma HSP90AA1 Predicts the Risk of Breast Cancer Onset and Distant Metastasis
Source: Front Cell Dev Biol. 2021 May 24;9:639596. doi: 10.3389/fcell.2021.639596 (PMC8181396; doi:10.3389/fcell.2021.639596)
Supplement: Supplementary file 8 [file Table_1.docx]

***Supplementary Material***

**
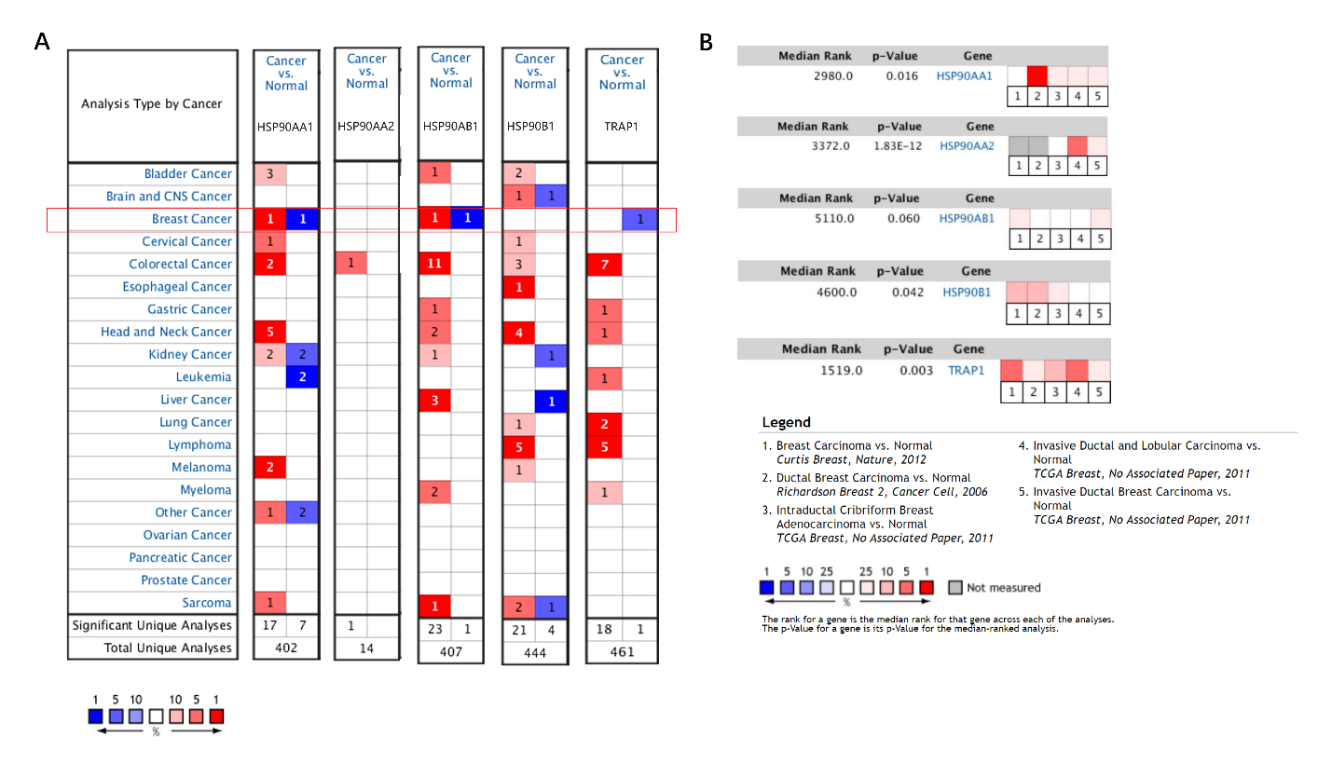
**

**Supplementary Figure 1. Heat shock protein 90 kDa family gene analysis in breast cancer (Oncomine database).** (A) The number of up-regulated datasets was found to be greater than the number of down-regulation datasets for most HSP90s, except for HSP90AA2, in various types of tumors compared with normal tissues. Red: over-expression; blue: down-regulation. (B) HSP90AA1, HSP90AA2, HSP90AB1, HSP90B1, and TRAP1 were significantly upregulated in five datasets, including TCGA.


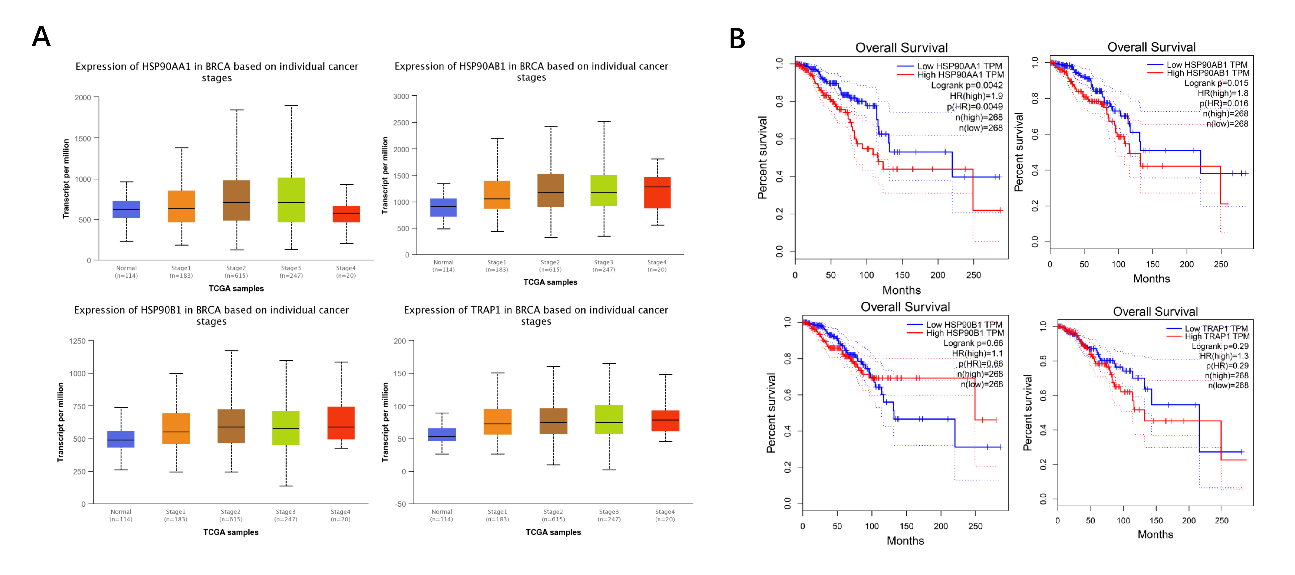


**Supplementary Figure 2. Correlations of the expression levels of HSP90s with tumor stages and prognostic values of HSP90s in breast cancer patients.** (A) The UALCAN is used to determine the relationship between the expression levels of *HSP90AA1, HSP90AB1, HSP90B1*, and *TRAP1* and tumor stages in breast cancer patients (p < 0.05). The x-axis indicates the stage of breast cancer tumors, and the y-axis shows the expression level of HSP90s (gene expression ~ pathological stage). (B) The overall survival curves comparing breast cancer patients with high (red) and low (blue) HSP90s expression levels are plotted using the GEPIA**.**

**
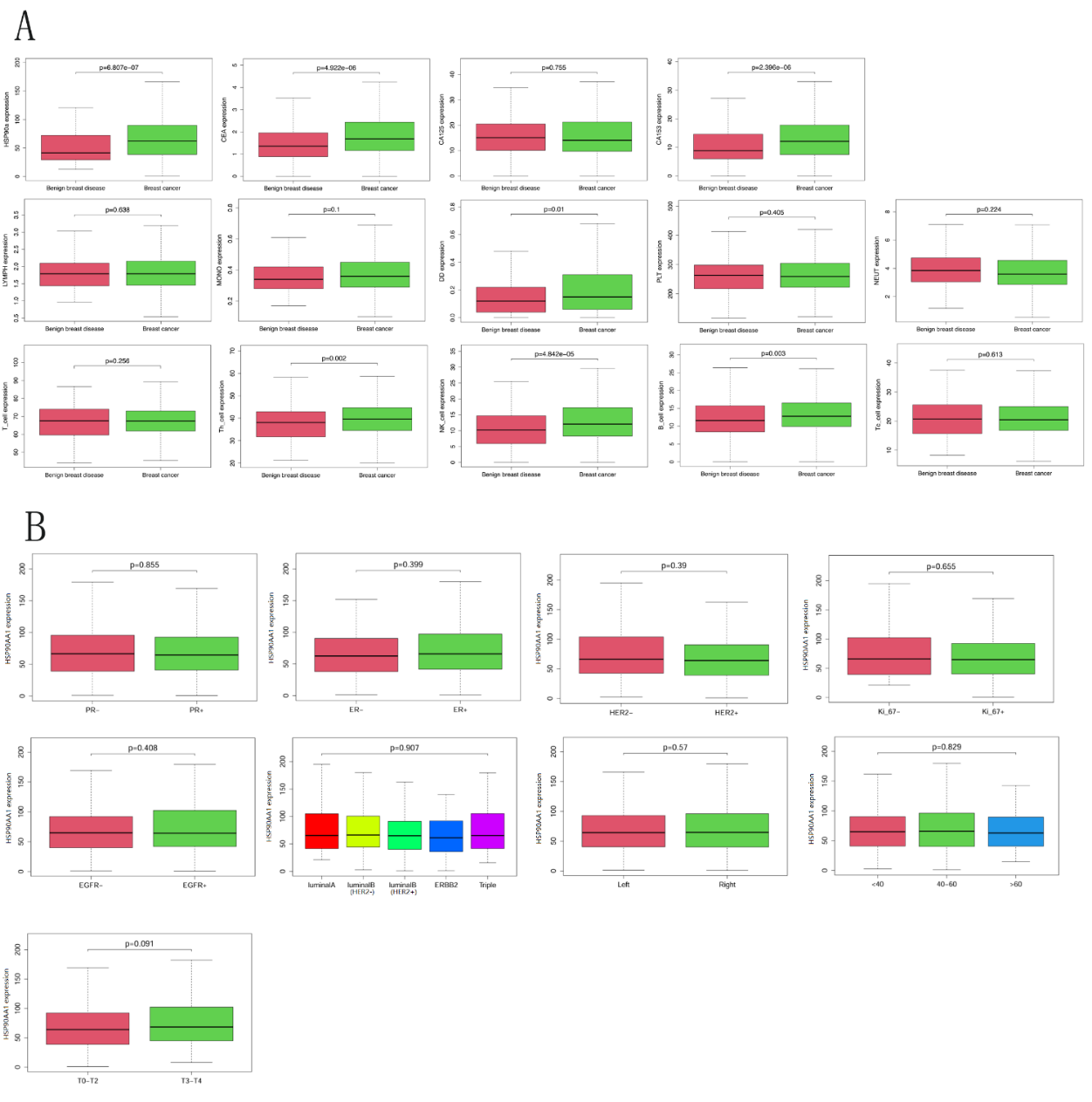
**

**Supplementary** **Figure 3. Boxplots of the cancer risk and metastasis risk for the different cohorts.** (A) Boxplots of clinical indicators in benign breast disease and in stage I, II, and III breast cancer. (B) Boxplots of HSP90AA1in different breast cancer patients. The clinical indicators analyzed using immunohistochemical analysis include: ER, PR, HER2, Ki-67, CK5/6, and EGFR; molecular types of breast cancer (luminal A, luminal B(HER2-), luminal B(HER2+), ERBB2, triple; the left and right breasts; and age. Differences between groups were estimated using Mann–Whitney U or Kruskal–Wallis tests, as appropriate.

**
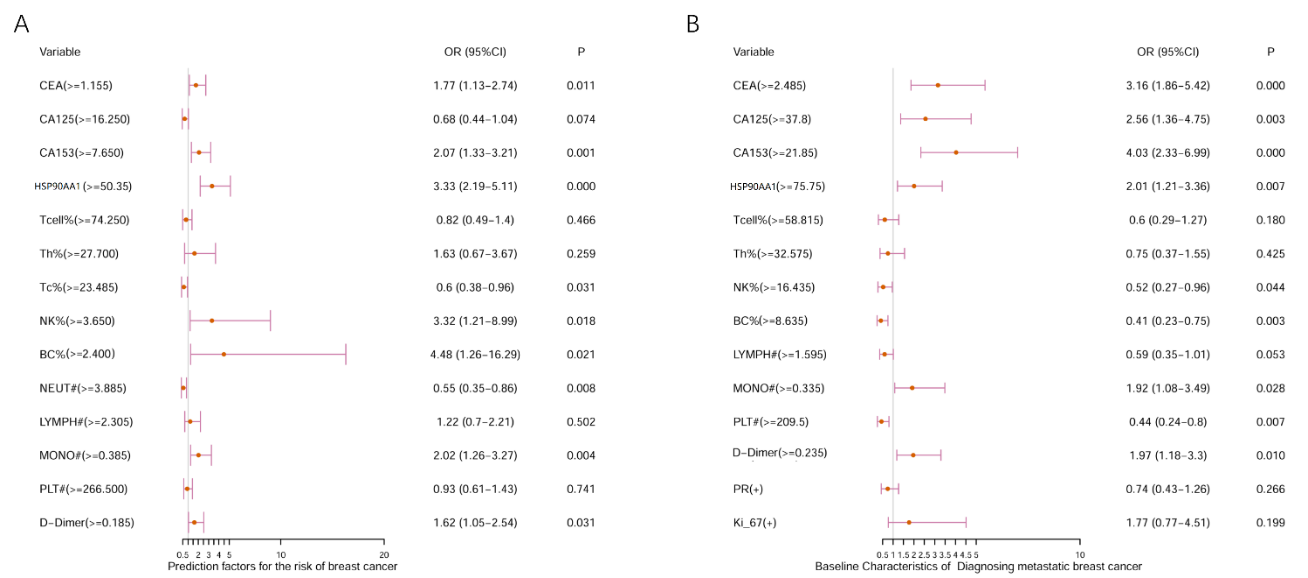
**

**Supplementary Figure 4. Prediction factors for our research.** (A) Cancer risk cohort, (B) The metastasis risk cohort.


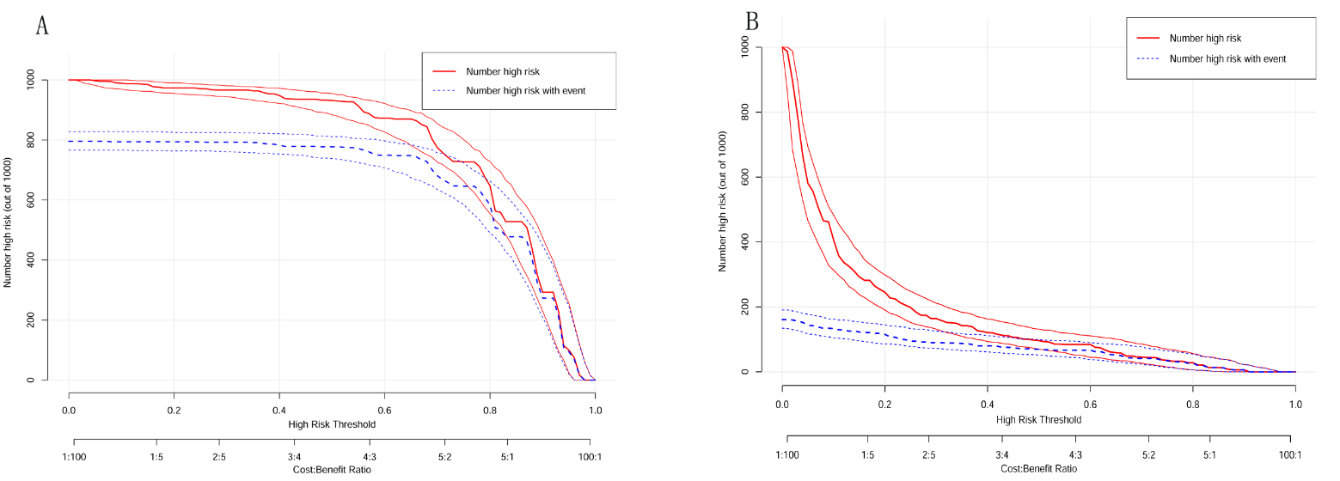


**Supplementary Figure 5. Clinical impact plots of the risk prediction model.** Of 1000 patients, the red curve represents the number of people who are classified as positive (high risk) by the simple model at each threshold probability, The blue curve shows how many of those would be true positives (cases). (A) Cancer risk cohort; (B) the metastasis risk cohort.


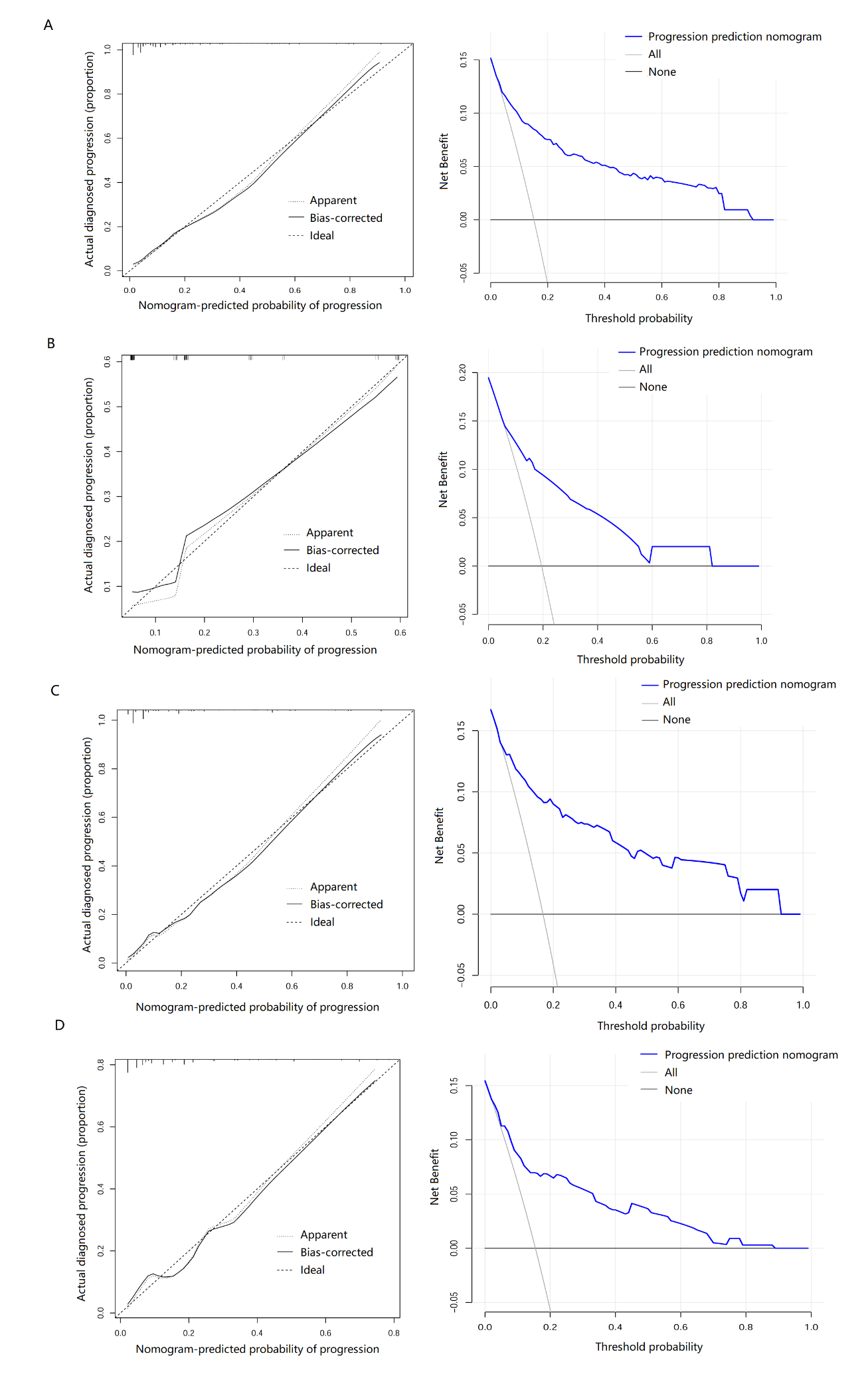


**Supplementary** **Figure 6. Calibration curves of the nomogram prediction in patients with different molecular types metastasis risk.** HR (A) positive, (B) negative, HER2 (C) positive, and (D) negative patients**.**


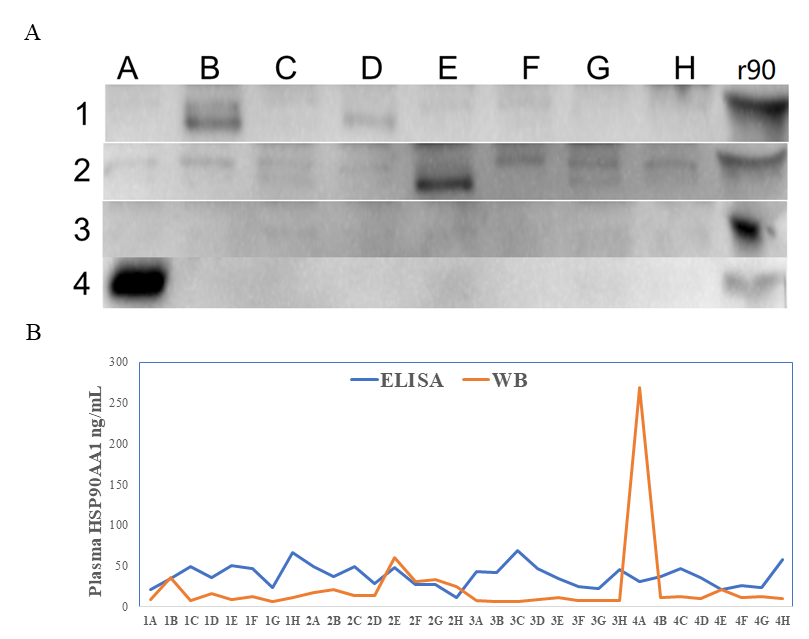


**Supplementary** **Figure 7. Western blotting (WB) methods verification levels of plasma Hsp90AA1 from partial ELSIA negative patients.**  (A) Detect plasma Hsp90AA1 in ELISA negative breast cancer patients by Western blotting. r90, recombinant Hsp90AA1 (75 ng/mL). (B) Plasma Hsp90AA1 concentration profiles categorized in ELISA negative patients with breast cancer: ELISA and WB.
